# Epidemiological and Molecular Characterization of Human Metapneumovirus (HMPV) in Children Under 14 Years of Age in Shijiazhuang City, China

**DOI:** 10.3390/pathogens15090909

**Published:** 2026-08-28

**Authors:** Sihan Li, Caixiao Jiang, Minghao Geng, Nana Guo, Wentao Wu, Ziyi Pang, Xianlei Zhou, Jingyi Xue, Yaojia Bian, Jiuqi Jia, Xu Han, Yan Li, Qi Li

**Affiliations:** 1 School of Public Health, Hebei Medical University, No. 361, Zhongshan East Road, Shijiazhuang 050017, China; 13231115057@163.com (S.L.); 17391003324@163.com (J.X.); 25031100085@stu.hebmu.edu.cn (Y.B.); 2 Hebei Provincial Center for Disease Control and Prevention, No. 97, Huai’an East Road, Shijiazhuang 050021, China; jiangcaixiao@163.com (C.J.); gmhcdc@126.com (M.G.); yufeiwet@163.com (N.G.); wuwentao@zju.edu.cn (W.W.); 17332326072@163.com (Z.P.); 18132255468@163.com (X.Z.); 15830953978@163.com (J.J.)

**Keywords:** HMPV, epidemiology, whole genome sequencing, respiratory infections, children

## Abstract

**Objective:** The objectives of this study were to analyze the epidemiological characteristics and whole-genome features of human metapneumovirus (HMPV) among children under 14 years of age in Shijiazhuang during 2022–2025, enrich the whole-genome data of HMPV in China, and provide a basis for further understanding the genetic diversity of the virus and for its prevention, control, and transmission intervention. **Methods:** A total of 1920 cases of acute respiratory infection were enrolled from three sentinel hospitals in Shijiazhuang. Nucleic acid screening was performed using real-time PCR. Whole-genome sequencing was conducted on 25 positive samples. Phylogenetic trees were constructed using the neighbor-joining method in MEGA, and nucleotide and amino acid sequence identity analyses were performed. **Results:** The overall detection rate of HMPV from 2022 to 2025 was 4.17%. A prominent peak in winter 2024 was the major distinguishing feature. Temporally, both influenza-like illness (ILI) and severe acute respiratory infection (SARI) cases showed marked seasonality during 2022–2025, with high-incidence periods in December–March and April–June. BLAST alignment and phylogenetic analysis showed that among the 25 sequenced HMPV specimens, genotype B2 was the most frequently identified genotype (13/25, 52%). **Conclusions:** HMPV epidemics in Shijiazhuang during 2022–2025 mainly occurred in winter–spring and spring–summer, with a notable detection peak in December 2024. The B2 genotype predominated among the sequenced HMPV isolates obtained in this study. The amino acid variation in the G gene was significantly higher than that in other genomic regions.

## 1. Introduction

Human metapneumovirus (HMPV) is an enveloped single-stranded negative-sense RNA virus belonging to the Pneumoviridae family, genus Metapneumovirus. Since its first identification and report in the Netherlands in 2001 [1], HMPV has become one of the important pathogens causing acute respiratory infections worldwide. HMPV infections predominantly affect children, the elderly, and immunocompromised individuals [2,3,4]. Its clinical manifestations are complex and similar to those of respiratory syncytial virus infection, ranging from asymptomatic or mild upper respiratory symptoms to bronchitis and pneumonia. In severe cases, acute respiratory distress syndrome may develop, which can be life-threatening [5,6]. The complete genome of HMPV is approximately 13 kb in length and contains eight genes encoding nine proteins, arranged from the 3′ to the 5′ end in the order N, P, M, F, M2, SH, G, and L [7]. HMPV is clearly classified into two major genetic lineages, A and B, which are further subdivided into subtypes A1, A2, B1, and B2. Subtype A2 can be additionally divided into three clusters: A2a, A2b, and A2c. Its antigenicity and genetic diversity play important roles in the infection, epidemic transmission, immune response, and prevention and control of the virus [8].

Currently, most data on the surveillance and research of HMPV in China come from a few large cities. Shijiazhuang is the capital city of Hebei Province and also a large population-gathering area in North China. The prevalence of local respiratory viruses has clear regional significance. However, in the post-pandemic era, complete investigations of HMPV infection among children under 14 years old or molecular epidemiological analyses in this region are still insufficient [9,10,11,12]. Therefore, the understanding of the actual epidemic pattern and genetic characteristics of HMPV in this region is still lacking. To make up for this deficiency, this study conducted PCR detection on a total of 1920 samples including throat swabs and bronchoalveolar lavage fluid collected from children under 14 years old in Shijiazhuang from 2022 to 2025. The results showed that there were 80 HMPV-positive cases. On this basis, combined with the description of epidemic characteristics and phylogenetic analysis, this study aims to clarify the epidemiological characteristics and genotype distribution of HMPV infection in children in Shijiazhuang and even Hebei Province, so as to provide a scientific basis for improving the local respiratory pathogen surveillance system, guiding clinical prevention and control, and promoting the research and development of related vaccines.

## 2. Materials and Methods

### 2.1. Sample Sources

According to the experimental needs, the cases were divided into the following two categories:

a. Outpatient influenza-like cases (ILI cases): Acute onset of illness (body temperature ≥ 38 °C), accompanied by either cough or sore throat, including nasal and pharyngeal swabs and other upper respiratory tract infection specimens.

b. National-level hospitalized severe acute respiratory infection cases (SARI cases): Hospitalized patients at the time of admission or within 48 h after admission, acute onset of illness, onset with history (temperature ≥ 38 °C), accompanied by cough, and a current onset of illness not more than 10 days, including alveolar lavage, sputum and other specimens of lower respiratory tract infections.

In this study, upper and lower respiratory tract specimens were collected from January 2022 to December 2025 at influenza surveillance sentinel hospitals (Hebei Provincial Children’s Hospital, the Fourth Hospital of Hebei Medical University, and Shijiazhuang Lujiazhuang Luquan Hospital), of which 20 ILI cases and 20 SARI cases were collected each month, for a total of 40 specimens per month, totaling 1920 specimens, which were stored in a −80 °C refrigerator for backup.

Age was stratified into four subgroups for epidemiological analysis: ≤1 year, >1 to ≤3 years, >3 to ≤5 years, and >5 to ≤14 years.

This stratification strategy was formulated according to the clinical and epidemiological characteristics of HMPV infection and local sentinel surveillance criteria. Children ≤ 1 year represent the infant group; children > 1 to ≤3 years and >3 to ≤5 years correspond to toddler and preschool-age children, respectively; and children under 5 years are well-established high-risk populations, as HMPV is the second most common pathogen causing acute lower respiratory tract infections in this population, with only respiratory syncytial virus having a higher prevalence. We further subdivided children under 5 years into three subgroups to capture epidemiological differences among infants, toddlers and preschool children. Children in the >5-to-≤14-year group cover school-age children, and 14 years is the upper age limit defined by our local sentinel surveillance inclusion criteria.

### 2.2. Main Reagents and Instruments

Nucleic acid extraction and purification reagents (Shanghai BioGerm Medical Technology., Ltd., Shanghai China); the Human Parvovirus Nucleic Acid Assay (Large Single Tube) Liquid Whole Fractionation Kit (Guangzhou Slin Medical Technology Co., Ltd., Guangzhou China); the RNA Pathogenic Microorganisms Library Capture Kit (iGeneTech Bioscience Co., Ltd., Beijing, China); and the QuantStudio 7 Flex Real-Time Fluorescence PCR System (Thermo Fisher Scientific, Waltham, MA, USA) were used.

### 2.3. Experimental Method

According to the instructions of the Rapid Nucleic Acid Extraction Kit, 200 μL of preservation solution of the specimen to be extracted was added to the 96-well plate of ① sample to extract viral nucleic acid. Real-time fluorescence quantitative PCR was performed according to the instructions and reaction conditions of the Human metapneumovirus Nucleic Acid Detection Kit. Results with a CT value ≤ 40 and obvious exponential growth were recorded as positive. The remaining nucleic acid was temporarily put into a −80 °C refrigerator for freezing and storage. The primers and probes of this commercial real-time PCR kit target highly conserved genomic regions of human metapneumovirus, designed to cover both genotype A and B. All clinical specimens in this study were tested using the same batch of reagents following unified experimental protocols. Although the manufacturer has verified the analytical sensitivity of the kit, dedicated validation experiments comparing amplification efficiency between genotype A and B were not provided by the manufacturer.

### 2.4. Whole-Genome Sequencing of HMPV

HMPV-positive nucleic acid samples with a CT value of ≤32 were selected for whole-genome sequencing. Using HMPV-positive RNA as a template, we sequentially completed fragmentation, reverse transcription, double-stranded cDNA synthesis, junction ligation and pre-PCR amplification to construct a hybridization capture pre-library. HMPV genome-specific probes TargetSeq^®^ DNA Probes(iGeneTech Bioscience Co., Ltd., Beijing, China) were used for liquid-phase hybridization capture, and the target sequences were enriched for post-capture PCR amplification. After the amplification products were purified by magnetic beads and passed double quality control, the libraries were diluted according to the platform requirements and uploaded to the Illumina High-Throughput Sequencing platform(illumina Inc., San Diego, CA, USA) for sequencing.

### 2.5. Analysis of Sequencing Results

After sequencing was completed, the sequencing results were sequenced on the National Center Biotechnology Information (NCBI) for sequence comparison and the published HMPV sequences of human metapneumovirus were downloaded, and 25 strains were screened as reference strains for phylogenetic analysis, and the phylogenetic tree was constructed by using the neighbor-joining method of the MEGA12 software (Neighbor-Joining, NJ), and the bootstrap value was set to 1000.

### 2.6. Statistical Analysis

Data were analyzed using R 4.4.1. For comparisons of detection rates between groups, the chi-square test was applied. For the temporal distribution analysis across month–year strata with several cells having expected counts < 5, a Monte Carlo-based chi-square test was performed (20,000 replicates, set. seed = 123), and standardized residuals were calculated to identify cells with prominent deviation. A two-sided *p* < 0.05 was considered statistically significant.

## 3. Results

### 3.1. Detection Rate and Epidemiological Characterization of HMPV

**Figure 1:**
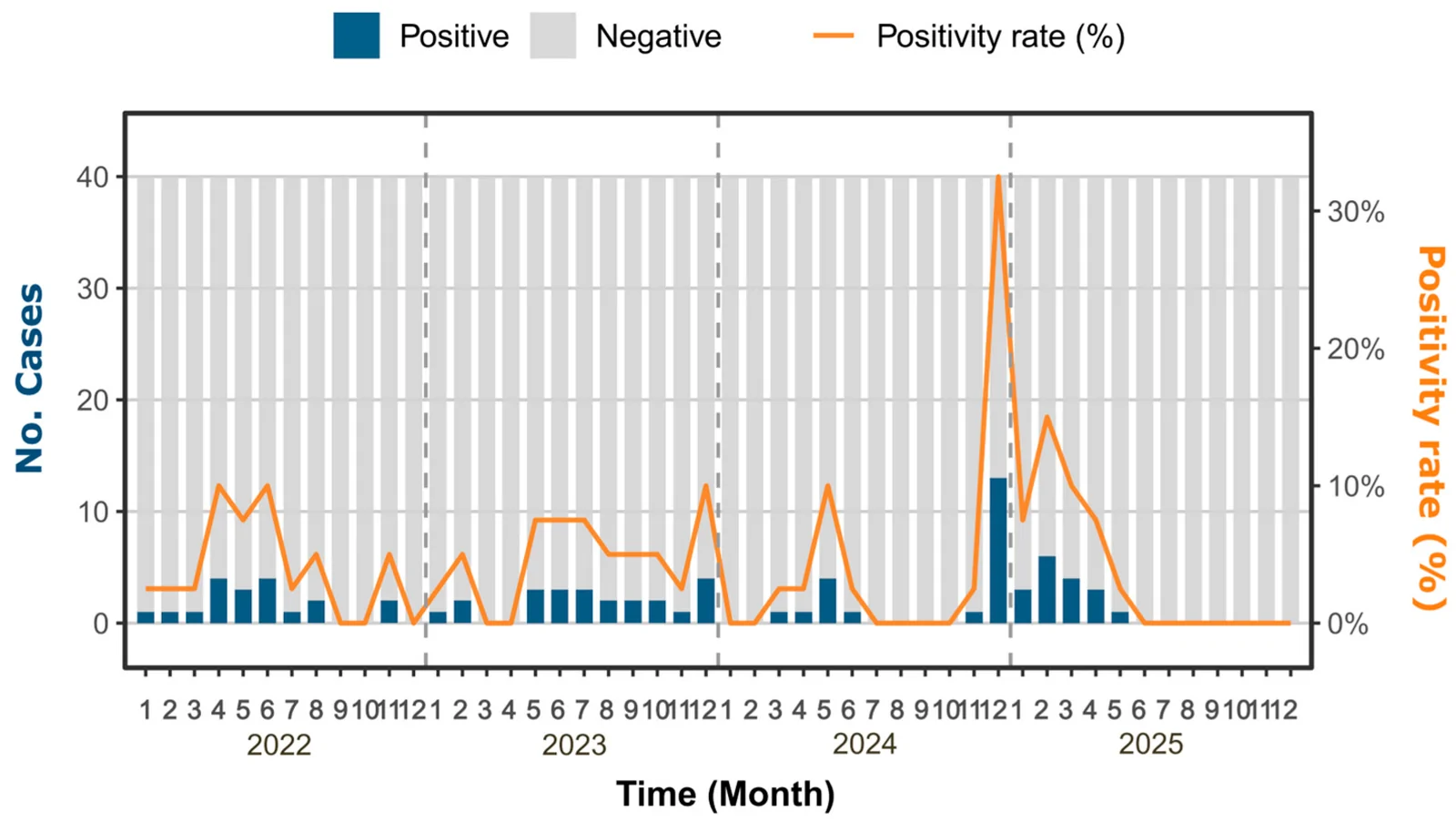
Trends in positive detection rates for HMPV, 2022–2025. Note: Dotted lines separate different calendar years.

During 2022–2025, a total of 1920 specimens were collected in this study, including 982 male patients and 938 female patients, and 80 positive samples were detected by PCR, with a total detection rate of 4.17%, of which 4.28% (42/982) was detected in male patients and 4.05% (38/938) in female patients, and the chi-square test showed that *p* > 0.05 (Table 1), i.e., the difference between the two was not statistically significant; the detection rates in 2022–2025 were 3.96% (19/480), 4.79% (23/480), 4.38% (21/480), and 3.54% (17/480), respectively, with no statistically significant difference in the detection rates in different years ($\chi^{2}$ = 1.04, *p* > 0.05). The Monte Carlo-based chi-square test (20,000 replicates, set.seed = 123) revealed an overall statistically significant temporal clustering of HMPV-positive cases ($\chi^{2}$ = 150.68, *p* < 0.001). Standardized residual analysis indicated that December 2024 contributed the most to the overall heterogeneity, representing the major epidemic peak during our study period. In terms of age distribution, the detection rate was the highest in the 3–5-year age group (6.67%), and the difference in detection rates among different ages was statistically significant ($\chi^{2}$ = 8.902, *p* < 0.05, Table 1). According to the mean trend of monthly positive rates, winter–spring and spring–summer were identified as the main high-incidence seasons of HMPV (Figure 1), indicating obvious temporal clustering of cases.

Among 960 children with ILI, there were 500 males and 460 females, and 52 positive samples were detected, with a detection rate of 5.42%, 31 males and 21 females in the positive samples, and the difference between the detection rates of males and females was not statistically significant ($\chi^{2}$ = 0.951, *p* = 0.330); 22 positive detections were detected in children between the ages of 3 and 5 years old, which accounted for 42.31% of the positive cases of ILI (22/52) (Table 2).

Among 960 SARI children, 482 were male and 478 were female, with a total of 28 positive samples detected, a detection rate of 2.92%, 11 males and 17 females in the positive samples, and the difference between the detection rates of males and females was not statistically significant ($\chi^{2}$ = 0.963, *p* = 0.326); 13 cases were detected in children aged 5–14 years old, which accounted for 46.43% (13/28) of all SARI-positive cases (Table 2).

**Figure 2:**
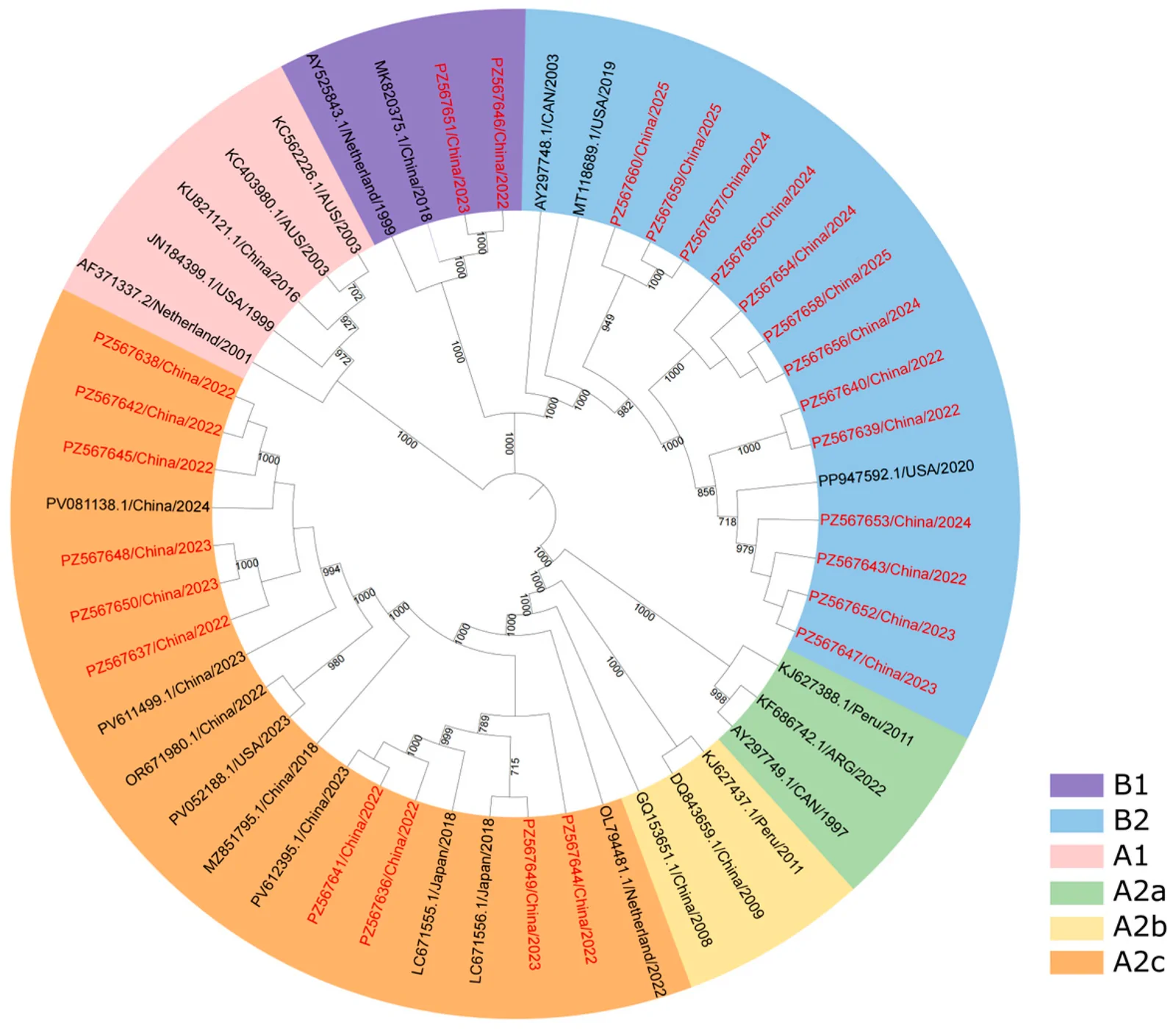
Evolutionary tree constructed based on the whole genome. Note: A phylogenetic tree was constructed using the neighbor-joining method; nodes with a bootstrap value (based on 1000 bootstrap replications) ≥70% are marked; equal-length branches were used for visualization, and only the dendrogram-based clustering relationships are shown. Different colors represent different genotypes, with local strains from Shijiazhuang indicated in red.

Thus, the detection rate in the ILI group was significantly higher than that in the SARI group ($\chi^{2}$ = 7.513, *p* < 0.05). There was no statistically significant difference in the overall age distribution of positive children between the two groups ($\chi^{2}$ = 6.747, *p >* 0.05). The positive detection rate in the 3–5-year age group of the ILI group (9.21%) was significantly higher than that of the SARI group (3.57%), which was the main source of the difference. In terms of temporal distribution, both ILI cases and SARI cases from 2022 to 2025 showed obvious seasonal fluctuations, with different epidemic peak periods in different years. ILI cases were mainly concentrated in May–June and December, with high-incidence periods in winter–spring (December–March) and spring–summer (April–June) and relatively low activity in summer–autumn (July–November). The peak of SARI cases was basically synchronized with that of ILI cases, but the overall positive detection rate in the SARI group was lower (Figure 2).

### 3.2. Phylogenetic and Homology Analysis

**Figure 3:**
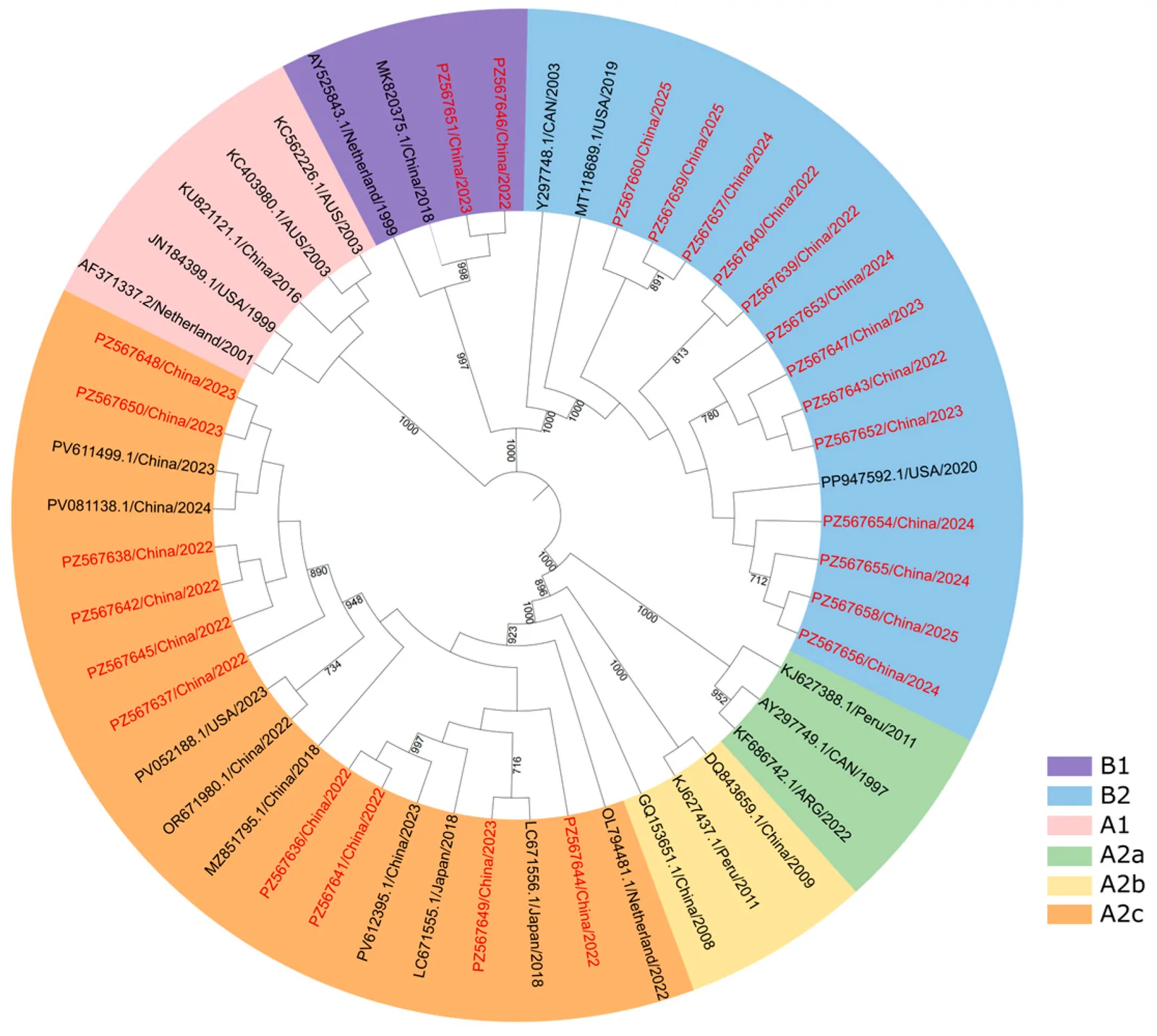
Evolutionary tree constructed based on F gene. Note: A phylogenetic tree was constructed using the neighbor-joining method; nodes with a bootstrap value (based on 1000 bootstrap replications) ≥70% are marked; equal-length branches were used for visualization, and only the dendrogram-based clustering relationships are shown. Different colors represent different genotypes, with local strains from Shijiazhuang indicated in red.

**Figure 4:**
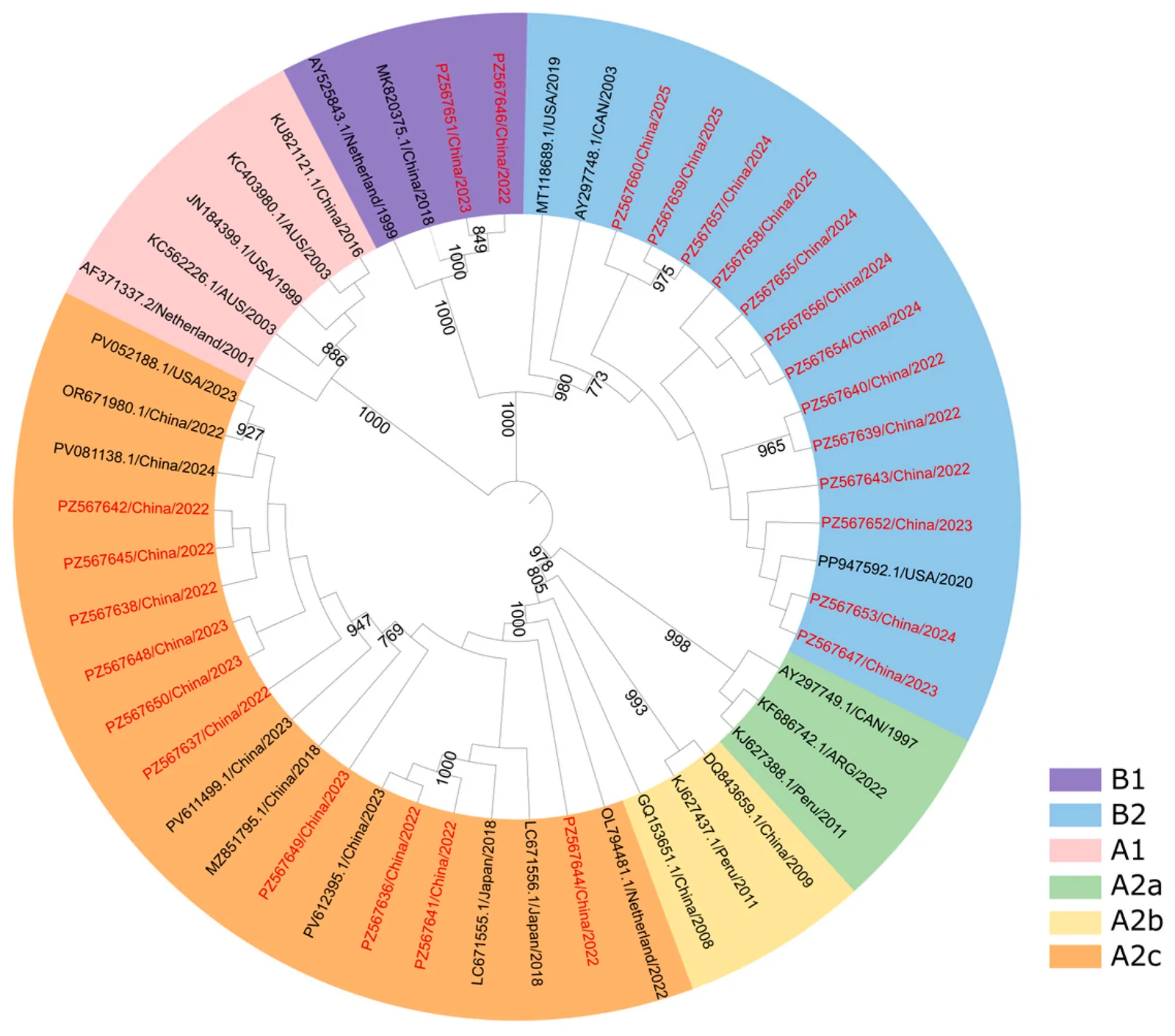
Evolutionary tree constructed based on G gene. Note: A phylogenetic tree was constructed using the Neighbor-Joining method; nodes with a bootstrap value (based on 1000 bootstrap replications) ≥70% are marked; equal-length branches were used for visualization, and only the dendrogram-based clustering relationships are shown. Different colors represent different genotypes, with local strains from Shijiazhuang indicated in red.

Whole-genome sequencing was performed on the samples with lower Ct values (Ct value ≤ 32). After sequencing, the whole-genome sequences of 25 HMPV strains were successfully obtained, and the sequence length of 22 strains was 13,250~13,432 nt. The sequence length of the other three strains is 13,020–13,050 nt, only the non-coding region at the 5′ end of the genome is partially deleted, and the coding regions of N, P, M, F, M2, SH and G structural genes are complete, and only a few sequences at the end of the L polymerase gene are not completely amplified, which does not affect the typing and the analysis of G and F gene variation. The whole-genome sequences of 25 HMPV strains obtained in this study have been uploaded to the GenBank database, and the Genbank accession numbers are PZ567636-PZ567660 in turn(Detailed sequence information is provided in Supplementary Materials); 25 whole-genome sequences of different strains of different types, years and regions were downloaded from NCBI to serve as the reference strains, which were compared with the whole-genome sequences of the 25 successful HMPV strains of human metapneumovirus. The phylogenetic tree was constructed using the neighbor-joining method to analyze the homology and evolutionary relationship, and the results showed that (Figure 2), among the 25 strains, there were 2 B1, 13 B2, and 10 A2c strains, respectively, of which B2 was the most numerous, which was in line with the results of genotyping by PCR. The sequenced subset suggested a possible temporal change from A2c to B2 during 2022–2025. Among the 10 A2c strains, 90% (9/10) harbored a 111-nucleotide repeat sequence in the G gene. To verify the reliability of the typing results, an evolutionary tree was constructed based on the highly conserved F gene and the highly variable G gene, respectively (Figure 3 and Figure 4), and the results obtained were consistent with the genome-wide evolutionary tree, which further confirmed the accuracy of the typing results of this study.

### 3.3. Sequence Consistency Analysis

**Figure 5:**
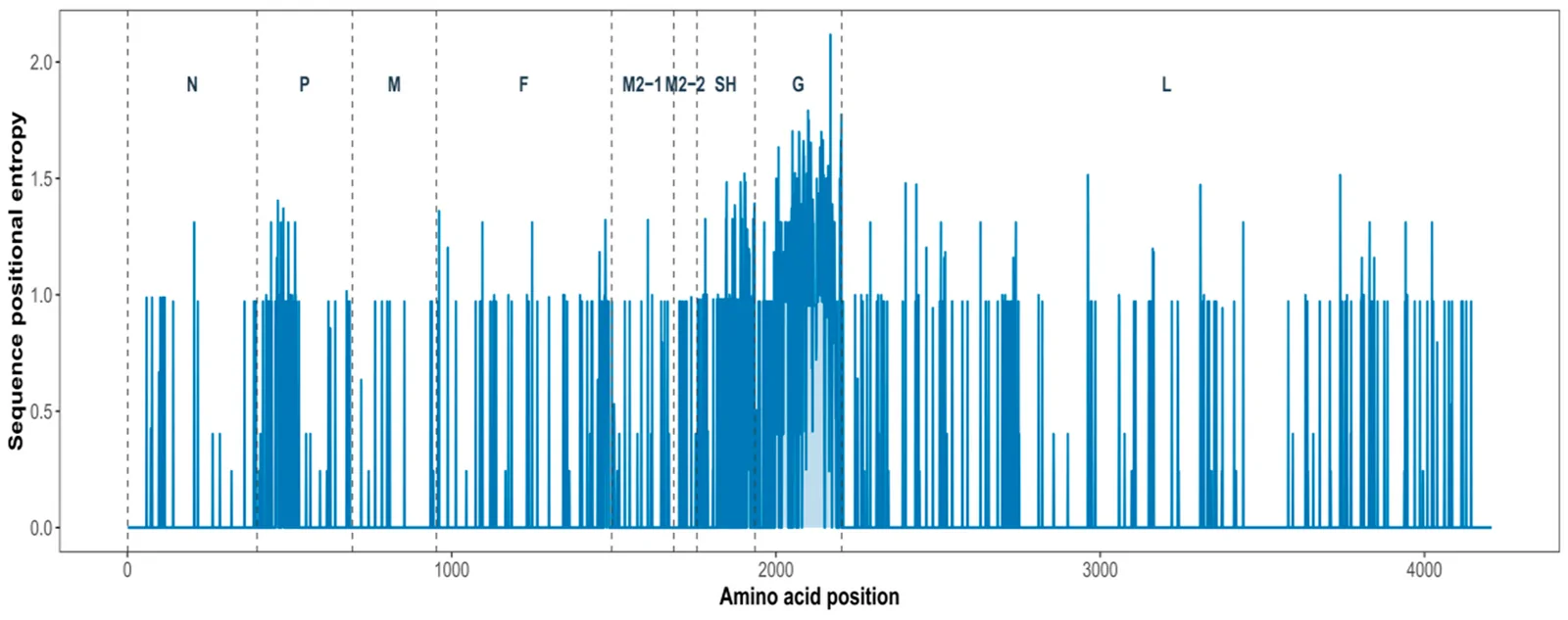
Site Position Entropy (SPE) analysis of amino acid residues in the protein-coding regions of HMPV. Note: The x-axis indicates amino acid positions along the concatenated HMPV polyprotein. The y-axis represents sequence positional entropy; higher entropy values correspond to greater amino acid variability at the given site. Vertical dashed lines denote boundaries of individual HMPV protein-coding open-reading frames (N, P, M, F, M2-1/M2-2, SH, G, L).

Nucleotide and amino acid sequence identity analyses were performed on the sequences. The nucleotide-level identity for whole-genome sequences ranged from 0.813 to 0.999. At the amino acid level, sequence identities differed substantially among individual HMPV genes, spanning 0.300 to 1 across all coding regions (Table 3). Shannon entropy analysis was used to calculate the Site Position Entropy (SPE) for each residue position of the HMPV amino acid sequences isolated in Shijiazhuang. The plotted results revealed substantial disparities in variation levels across different sites of the HMPV amino acid sequences. Most protein-coding regions exhibited low SPE values, indicating high conservation. In contrast, amino acid residues encoded by the G gene showed high SPE values, demonstrating that the G gene-encoded region harbors markedly greater amino acid variation than other genomic regions (Figure 5).

## 4. Discussion

This study conducted an epidemiological investigation and statistical analysis of human metapneumovirus among children under 14 years old in Shijiazhuang from 2022 to 2025. Through whole-genome sequencing of 25 samples, the genotype distribution and evolutionary relationship of HMPV in this region were preliminarily demonstrated. The results showed that [13,14] HMPV infection exhibited obvious changes before and after the adjustment of COVID-19 prevention and control measures and gradually returned to the characteristic of high incidence in winter and spring in the post-pandemic era. These findings provide a certain reference value for understanding the epidemic pattern of respiratory pathogens in the post-pandemic era.

In this study, the overall detection rate of HMPV in children with acute respiratory infection was 4.17%. In terms of temporal distribution, the detection rate in December 2024 was significantly higher than in other months. This trend was generally consistent with several studies in Hebei Province and other parts of China [15,16]. A retrospective study in Turkey covering 2021 to 2024 showed that the number of HMPV cases peaked in 2022 and then decreased markedly in 2023–2024 [17]. Surveillance data from Washington State also indicated that the seasonality of HMPV was disrupted in 2020 and returned to a normal seasonal pattern after 2023 [18]. Many scholars have attributed this phenomenon to the “immunity debt” effect caused by the long-term implementation of non-pharmaceutical interventions (NPIs) during the COVID-19 pandemic. That is, prolonged lack of exposure to common respiratory pathogens among children led to insufficient immune stimulation and reduced population immunity, resulting in a compensatory epidemic peak after the adjustment of prevention and control measures [19,20].

The seasonal pattern of HMPV also changed around 2023. Before the COVID-19 pandemic, influenza peaks in northern China generally occurred in winter and spring. However, in this study, HMPV epidemics during 2022–2023 mostly appeared in spring and summer. Studies in some southern regions also observed that the epidemic season changed significantly after the lifting of non-pharmaceutical interventions in 2023 [21,22]. This suggests that long-term prevention and control measures may have temporarily reshaped the seasonal epidemic patterns of HMPV and other respiratory viruses. During the transition from this altered pattern back to the traditional winter–spring epidemic pattern, relevant medical institutions should re-evaluate the active epidemic periods of viruses such as HMPV when formulating respiratory prevention and control strategies, so as to avoid relying on previous perceptions to determine prevention priorities and prevent surveillance blind spots.

In terms of age distribution, the proportion of HMPV-positive children in this study was the highest in the 3–5-year age group, accounting for 36.25% of all positive cases. This result is generally consistent with relevant statistics from a multicenter study published in Hebei Province during the same period. The related literature has pointed out that after 2019–2020, children aged 3–5 years (accounting for 40.93%) replaced children under 3 years as the main infected population [23]. A related study in Wenzhou also reported a similar changing trend. It is speculated that the shift in the age distribution center to older children may be due to the stricter protection of infants and young children during the pandemic, whose immune systems failed to establish immunity through natural infection. However, as they grow older and enter kindergartens or schools, increased social contact raises the risk of infection. Infants aged ≤1 year may obtain partial protection from residual maternally transferred antibodies, which contributes to their relatively moderate positive rate. Children aged 1–3 years have largely waned maternal-derived immunity, yet many have not yet been enrolled in full-time childcare settings, leading to lower-intensity social exposure compared with 3–5-year-old preschoolers. This may partly explain why their detection rate was higher than that of older children but lower than the 3–5-year-old subgroup. Children aged 6–14 years have relatively mature immune systems and relatively high compliance, making it easier for them to develop epidemic prevention habits such as hand washing and mask wearing, thus resulting in a lower positive detection rate. A global meta-analysis showed that children under 5 years old remain the high-risk group for HMPV infection [24], indicating that age is still an important risk factor for HMPV infection. Our data further highlight that 3–5-year-old preschool children represent the key population driving community transmission, while infants ≤ 1 year and children aged 1–3 years also have non-negligible detection rates and should not be ignored in routine sentinel surveillance. Practically, targeted prevention and surveillance measures should be implemented in multiple scenarios. In home-care settings, caregivers should pay attention to respiratory hygiene to reduce intra-household transmission risk. For nurseries and kindergartens, morning health checks, timely isolation of symptomatic children and regular ventilation are encouraged to mitigate cluster transmission. Hospitals should maintain continuous sentinel surveillance for HMPV among pediatric respiratory patients, especially during epidemic seasons.

This study compared the detection rates of human metapneumovirus (HMPV) between severe acute respiratory infection (SARI) and influenza-like illness (ILI) specimens. The results showed that the HMPV detection rate was 5.42% among ILI cases and 2.92% among SARI cases; the detection rate in ILI cases was significantly higher than that in SARI cases. This finding is contrary to the results of a global meta-analysis, indicating that the positive proportions among outpatient and hospitalized cases may vary across regions and epidemiological contexts. Illnesses caused by HMPV infection can present as relatively mild influenza-like symptoms, as well as severe acute respiratory infections requiring hospitalization.

Genetic sequencing analysis was performed on positive samples with low CT values. After phylogenetic tree construction by aligning sequencing results with reference strains for genotyping, the results demonstrated that among the 25 HMPV strains sequenced in this study, subtype B2 was the most frequently identified genotype, accounting for 52% (13/25), followed by subtype A2c at 40% (10/25). A total of 10 A2c strains were obtained in the present study, and 90% (9/10) of these strains harbored a 111-nucleotide (nt) repeat sequence within the G gene. Regarding temporal distribution, the detected strains during 2024–2025 were predominantly of the B2 subtype, whereas during 2022–2023, the majority consisted of the A2c subtype. The sequenced subset suggested a possible temporal shift from A2c to B2 among our sampled isolates, which is consistent with findings from multiple previous domestic and international studies [25,26]. Our entropy analysis revealed high variability within the G protein, consistent with its role in immune evasion. This molecular plasticity contrasts with the high conservation of the F protein. Therefore, while the G gene is useful for tracking epidemiological shifts (such as the A2c to B2 transition observed in our sequenced subset here), the conserved F protein remains the superior target for developing broad-spectrum vaccines and monoclonal antibodies.

In recent years, surveillance and investigations on the genotypic dynamics of HMPV have been carried out across multiple provinces and municipalities in China. A study conducted in Hangzhou during 2022–2023 reported that A2.2.2 was the dominant local genotype, representing an obvious shift from subtype B2 prevalent in 2020–2021 [27]. Subtype B2 remained predominant in Beijing from 2014 to 2024 [28]. Another Beijing-based study covering 2017–2019 revealed that 92.59% of HMPV sequences belonged to subtype A2b1, and 96% of A2b1 sequences contained the 111-nt repeat in the G gene; such repetitive structures may facilitate immune evasion [29]. Internationally, researchers from the United States further proposed an evolutionary model for the A2b2-111 sublineage, suggesting that this sublineage may have evolved from the A2b2-180 variant via a 69-nt deletion [18]. Accumulating evidence also indicates that HMPV worldwide undergoes selective evolution analogous to human respiratory syncytial virus (hRSV) [30]. Subtype A2.2.2 strains have also been detected in Saudi Arabia in recent years [31]. Furthermore, a Japanese study confirmed that HMPV subtypes A2b2, A2.2.2 and A2c refer to the same subtype under different nomenclature systems. These findings highlight the global genetic diversity and inconsistent nomenclature of HMPV.

This study was a retrospective analysis conducted based on subjects recruited from three hospitals in Shijiazhuang City. Although the enrolled institutions included major specialized children’s hospitals in the local area, community cases were not incorporated, which may lead to certain selection bias. In addition, the study period covered the year 2022 during the COVID-19 pandemic. Changes in medical-seeking behaviors and pathogen detection strategies during this period may have exerted a certain impact on the study results. Furthermore, only HMPV-positive specimens with Ct ≤ 32 were selected for whole-genome sequencing, and 25 complete sequences were finally obtained. The genotype distribution derived from these sequenced isolates may not fully represent all HMPV-positive cases enrolled in our study, and genotypic findings should be interpreted with caution. Further studies with larger sequencing sample sizes including specimens with higher Ct values are needed to verify the circulating characteristics of HMPV in this region. In addition, clinical metadata including fever duration and complication information were not systematically collected in this study, which restricts our ability to compare clinical manifestations between different subtypes. Future work should incorporate comprehensive clinical data to explore potential clinical differences among circulating subtypes.

## 5. Conclusions

This study preliminarily characterized the epidemiological prevalence and genotypic distribution of human metapneumovirus (HMPV) among children aged under 14 years in Shijiazhuang City from 2022 to 2025. The analytical results indicated that the epidemic trend of HMPV changed significantly following the adjustment of COVID-19 prevention and control measures in the post-pandemic era, which was speculated to be associated with the relaxation of non-pharmaceutical interventions (NPIs). The HMPV detection rate was remarkably higher in the influenza-like illness (ILI) group than in the severe acute respiratory infection (SARI) group, suggesting that HMPV positivity is relatively common among outpatient children, thereby emphasizing the necessity of strengthening HMPV surveillance in pediatric outpatient settings. Genetic sequencing results from our sequenced subset preliminarily suggested a possible subtype shift from A2c to B2 among our sampled isolates, with B2 being the most frequently identified genotype among the sequenced specimens. Collectively, our study provides practical baseline data for future HMPV surveillance among children in this region. Specifically, the annual positive rates, seasonal epidemic patterns, age-stratified detection profiles, and comparative results between ILI and SARI cases, as well as HMPV sequence data deposited in GenBank from our study, can serve as reference benchmarks for subsequent local sentinel and molecular epidemiological surveillance. Future work should include continuous long-term pathogen monitoring, molecular genotyping analysis of circulating HMPV strains, multicenter investigations, and further in-depth collection of clinical information to explore the association between HMPV infection and diverse disease outcomes among pediatric populations. Moreover, the epidemic peak observed in the winter of 2024 further indicates that continuous surveillance of respiratory pathogens including HMPV and optimized prevention and control strategies for pediatric respiratory infections are essential in the post-pandemic era.

## Figures and Tables

**Table 1:** Demographic characteristics of positive specimens in 2022–2025.

Category	Number of Cases (n)	Number of Positive Cases (n)	Prevalence of HMPV (%)	*p* Value
Gender				0.805
Male	982	42	4.28	
Female	938	38	4.05	
Age (years)				0.0041
≤1	377	13	3.45	
>1 to ≤3	408	15	3.68	
>3 to ≤5	435	29	6.67	
>5 to ≤14	700	23	3.29	
Total	1920	80	4.17	

**Table 2:** Age distribution of HMPV-positive children among outpatient ILI and hospitalized SARI groups.

Age (Years)	≤1		>1 to ≤3		>3 to ≤5		>5 to ≤14	
	Cases	Positive (%)	Cases	Positive (%)	Cases	Positive (%)	Cases	Positive (%)
ILI	244	9 (3.69%)	226	11 (4.87%)	239	22 (9.21%)	251	10 (3.98%)
SARI	133	4 (3.01%)	182	4 (2.20%)	196	7 (3.57%)	449	13 (2.90%)

Note: ILI is influenza-like illness; SARI is severe acute respiratory infection; HMPV is human metapneumovirus.

**Table 3:** Identity of 25 HMPV whole-genome sequences.

Gene	Nucleotide Sequence Identity		Amino Acid Sequence Identity	
	All the Sequences	Our Sequences	All the Sequences	Our Sequences
N	0.852–1	0.852–1	0.952–1	0.960–1
P	0.795–1	0.797–1	0.827–1	0.827–1
M	0.842–1	0.846–1	0.961–1	0.965–1
F	0.825–1	0.828–1	0.926–1	0.929–1
M2–1	0.839–1	0.844–1	0.925–1	0.936–1
M2–2	0.833–1	0.838–1	0.887–1	0.901–1
SH	0.685–1	0.711–1	0.563–1	0.612–1
G	0.577–1	0.577–1	0.300–1	0.324–1
L	0.835–1	0.838–1	0.935–1	0.935–1
Whole genome sequence	0.812–0.999	0.813–0.999	-	-

## Data Availability

The genome sequence of human metapneumovirus supporting the results of this study has been submitted to NCBI Genbank. The accession numbers are PZ567636 to PZ567660 (submission number: SUB16259879). These sequences have not been made public at present and will be made public when this manuscript is officially published.

## Supplementary Materials

The following supporting information can be downloaded at: https://www.mdpi.com/article/10.3390/pathogens15090909/s1.

## Abbreviations

HMPV	human metapneumovirus
ILI	influenza-like illness
SARI	severe acute respiratory infection
NCBI	National Center Biotechnology Information
SPE	Site Position Entropy
