# Supplementary material for: Epidemiological and Molecular Characterization of Human Metapneumovirus (HMPV) in Children Under 14 Years of Age in Shijiazhuang City, China
Source: Pathogens. 2026 Aug 28;15(9):909. doi: 10.3390/pathogens15090909 (PMC13610085; doi:10.3390/pathogens15090909)
Supplement: Supplementary file 1 [file pathogens-15-00909-s001.zip › Genbank Submission Record.pdf]

GenBank PZ567636-PZ567660

gb-admin@ncbi.nlm.nih.gov

收件人: LSH13231115057@126.com, 13231115057@163.com

时　间: 2026-6-23 19:25:20

附　件:

Dear GenBank Submitter:

Thank you for your submission of sequence data to GenBank, a contribution which will benefit the scientific community.

We have provided GenBank accession numbers for your nucleotide sequences:

SUB16259879 HMPV-SJZ-2022-01 PZ567636
SUB16259879 HMPV-SJZ-2022-02 PZ567637
SUB16259879 HMPV-SJZ-2022-03 PZ567638
SUB16259879 HMPV-SJZ-2022-04 PZ567639
SUB16259879 HMPV-SJZ-2022-05 PZ567640
SUB16259879 HMPV-SJZ-2022-06 PZ567641
SUB16259879 HMPV-SJZ-2022-07 PZ567642
SUB16259879 HMPV-SJZ-2022-08 PZ567643
SUB16259879 HMPV-SJZ-2022-09 PZ567644
SUB16259879 HMPV-SJZ-2022-10 PZ567645
SUB16259879 HMPV-SJZ=2022-11 PZ567646
SUB16259879 HMPV-SJZ-2023-01 PZ567647
SUB16259879 HMPV-SJZ-2023_02 PZ567648
SUB16259879 HMPV-SJZ-2023-03 PZ567649
SUB16259879 HMPV-SJZ-2023-04 PZ567650
SUB16259879 HMPV-SJZ-2023-05 PZ567651
SUB16259879 HMPV-SJZ-2023-06 PZ567652
SUB16259879 HMPV-SJZ-2024-01 PZ567653
SUB16259879 HMPV-SJZ-2024-02 PZ567654
SUB16259879 HMPV-SJZ-2024-03 PZ567655
SUB16259879 HMPV-SJZ-2024-04 PZ567656
SUB16259879 HMPV-SJZ-2024-05 PZ567657
SUB16259879 HMPV-SJZ-2025-01 PZ567658
SUB16259879 HMPV-SJZ-2025-02 PZ567659
SUB16259879 HMPV-SJZ-2025-03 PZ567660

The GenBank accession numbers should appear in any publication that reports or discusses these data, as it gives the community a unique label with which they may retrieve your data from our on-line servers. You may prepare and submit your manuscript before your accessions are released in GenBank.

Submissions are not automatically deposited into GenBank after being accessioned. Each sequence record is individually examined and processed

by the GenBank annotation staff to ensure that it is free of errors or problems.

You have requested that your data are to be held confidential until:

Jan 1, 2027

They will not be released to the public database until this date, or until the data or accession numbers appear in print, whichever is first.

Since the flatfile record is a display format only and is not an editable format of the data, do not make changes directly to a flatfile. For complete information about different methods to update a sequence record, see: https://www.ncbi.nlm.nih.gov/Genbank/update.html

Any inquiries about your submission should be sent to gb-admin@ncbi.nlm.nih.gov

For more information about the submission process or the available submission tools, please contact GenBank User Support at info@ncbi.nlm.nih.gov.

Please reply using the current Subject line.

Sincerely,

GBC-222 1
Contractor

The GenBank Direct Submission Staff
Bethesda, Maryland USA
****
gb-admin@ncbi.nlm.nih.gov (for updates/replies to GenBank entries)
info@ncbi.nlm.nih.gov (for general questions regarding GenBank)
****
